# Clinical Aspects of Hemilesions of the Cord

**Published:** 1918

**Authors:** 


***Clinical Aspects of Hemilesions of the Cord.*** Leading Article, *N. Y. Med. Jour.* Dec. 22, 1917.

In this war medullary hemisections, properly speaking by sharp edged instruments are relatively rare, while pathological lesions are rather frequent. Severe, direct, or indirect traumatism, e. g., fracture of the vertebrae with bone splinters, dislocation of the spine, with or without fracture and incomplete dislocation, are the causative factors.

Sudden inflexion of the spine from some violence, or its forced rotation, falls upon the knees, from a considerable height, or on the feet or buttocks, or a heavy weight falling some distance and striking the spine, may result in hemilesions of the cord.

Other conditions which may result in the Brown-Sequard syndrome are : periostitis, osteitis, arthritis, exostoses, enchrondromata, extra- or intrameningeal haemic collections whether the blood be coagulated or not, the myelitides and various meningitides, the late lesions of lues, benign and malignant neoplasms, hematomyelia, aortic aneurysm which has eroded the spine, etc., etc. All reported cases of those not experimentally produced are comparable. In hemilesion a double symptom dominates, viz : a motor hemiparesis with hyperaesthesia of the corresponding side and a superficial hemi-anaesthesia with preservation of movement on the opposite side.

The sensorimotor disturbances can be classified under four headings :

(1) Motor paralysis limited to the lower limb on the side of the hemisection. Sensory disturbances do not reach the root of the limo. The Cord is involved below the level of the ninth dorsal vertebra.

(2) Motor paralysis and sensory troubles extend above the roots of the lower limbs and involve a portion of the trunk. Cord involved between the middle and upper dorsal vertebrae.

(3) The upper limbs and thorax participate in the sensorimotor disturbances. Frequently get manifestations in relation to a paralysis of the great cervical sympathetic, and pneumogastric nerves. Cord involved at a level above the sixth cervical vertebra.

(4) Motor and sensory troubles invade an entire half of the body up to the face, the throat and the neck included. Cord involved at its upper portion just above the occipital protuberance. Importance is placed in the fact that in man, in traumatic hemisection of the cord Brown-Sequard's syndrome may only develop after a lapse of time while at the beginning a motor paraplegia, or even tetraplegia predominates.

(5) Hemilesions of the cord. Generally speaking, when the hemilesion does not involve the cord at its upper end, life is rarely endangered. Here the functional disturbances tend to improve progressively. Of the primary symptoms, paresis of some of the muscles, trembling of many, and slight changes in sensibility traces will be retained.

Microscopically, regeneration of the nerve elements has never been detected and in man return of functions has occurred in cases in which there has been no regeneration. This return of function is undoubtably due to compensation on the part of the cord which has remained intact or by an intercrossing of the motor fibres throughout the entire length of the cord.

